# Supplementary material for: Are carbon emissions trading and green financial instruments synergistic? -Comprehensive quantitative research based on content analysis
Source: PLoS One. 2024 Mar 7;19(3):e0298601. doi: 10.1371/journal.pone.0298601 (PMC10919601; doi:10.1371/journal.pone.0298601)
Supplement: S1 Data — (ZIP) [file pone.0298601.s001.zip › Data/地级市Z_F工作报告.zip!/╣π╬≈/╣≡┴╓╩╨/└┤╫╘╣1⁄2╓┌║┼í╛┬φ┐╦╩2╛▌═°í┐_2013.docx]

各位代表：

现在，我代表市人民政府向大会作工作报告，请予审议，并请各位政协委员和列席会议的同志提出意见。

2012年工作回顾

过去的一年，面对错综复杂的经济形势和改革发展稳定的艰巨任务，我们在自治区党委、政府和市委的领导下，在市人大、市政协的监督支持下，坚持稳中求进、好中求快的工作总基调，牢牢把握主题主线，全面实施“五大工程”，全市呈现出经济较快增长、民生不断改善、社会和谐稳定的良好局面。

初步统计，全年地区生产总值1492.05亿元，增长13.3%。农业增加值274.51亿元，增长6.6%；规模以上工业增加值494.81亿元，增长24.5%；第三产业增加值522.60亿元，增长9.2%。全社会固定资产投资1462.40亿元，增长28.2%。组织财政收入163.56亿元，增长15.2%。银行业金融机构各项贷款余额1053.15亿元，增长16.1%。城镇居民人均可支配收入22300元，增长12.2%；农民人均纯收入7328元，增长15.9%。城镇化率达41.8%。主要经济指标增幅好于预期，地区生产总值、第一产业总产值和增加值、规模以上工业总产值和增加值等多项指标增幅位居全区前列。

一年来，我们主要做了以下工作：

一、以“两化”融合推进工业转型升级，产业结构进一步优化

工业增速加快质量提高。全部工业总产值1821.59亿元、增加值583.03亿元，分别增长22.6%、19.3%，工业对经济增长的贡献率达58.3%。工业固定资产投资538.26亿元，增长45.3%。实施投资超亿元重点项目178项，占实施工业项目总数的三分之一，增长16.3%；实施新兴产业项目59项，增长63.8%。中化橡胶高等级子午线轮胎产业化一期工程、桂林福达10万吨精密锻件技改等一批重大项目竣工投产。优势和传统产业发展壮大，装备制造业、电子信息产业产值分别突破400亿元、200亿元，建材、包装与竹木加工成长为超百亿元产业。

“抓大壮小扶微”工程有效实施。大力扶持发展潜力大、科技含量高、就业带动效应明显的企业，新增产值10亿元以上企业5家、亿元以上企业70家、规模以上企业49家。加强对小微企业的创业扶持和融资服务，市中小企业服务中心成为全国中小企业公共服务示范平台，发放微型企业财政扶持资金额度位居全区前列，新增各类微型企业2085家，增长80%。

园区集聚效应显著增强。园区工业总产值956亿元，增长27.5%，占全部工业总产值比重超过50%。高新区技工贸总收入突破600亿元，增长24%；苏桥经济开发区工业总产值76亿元，增长48.5%；西城经济开发区工业总产值271.16亿元，增长28.3%。兴安工业集中区成长为百亿元园区；恭城、灌阳工业集中区成长为50亿元园区；雁山工业集中区被自治区确认为B类产业园区，一批特色园区加快形成。

信息化建设稳步推进。深入推进国家级“两化”融合试验区建设，新增自治区级信息化应用示范企业120家，总数达285家，居全区第二位。旅游、政务、公共管理信息化服务能力进一步提升，行业信息化应用深度发展。全市移动电话、互联网用户分别发展到446万户和74万户。网络与信息安全进一步加强。

二、以“两大改革”推动第三产业融合发展，服务业体系进一步完善

建设桂林国家旅游综合改革试验区，打造桂林国际旅游胜地取得重大突破。经国务院同意，《桂林国际旅游胜地建设发展规划纲要》获国家发改委批复，桂林发展上升为国家战略。自治区促进桂林国际旅游胜地建设的相关政策已研究上报，国际旅游胜地建设及宣传筹划工作全面启动，一批重大项目正在统筹实施。桂林世界旅游城规划体系基本形成，实施项目276项，完成投资197.36亿元。漓江5A级景区完成整改，独秀峰·王城景区进入国家5A级旅游景区行列，新增4A级景区3家。旅游目的地建设积极推进，我市被评为“2012年度国内最佳旅游目的地”，成为首批“全国旅游刷卡无障碍示范区”城市；阳朔入选“2012年中国十佳城市慢游地”，灌阳被评为“全国休闲农业与乡村旅游示范县”；会仙湿地被列入国家湿地公园试点《印象·刘三姐《梦幻漓江》入选全国文化旅游重点项目支持名录。接待游客突破3000万人次，达3292.65万人次，增长18.1%，旅游总收入276.87亿元，增长26.8%；入境游客达182.4万人次，增长11%，增幅居全国主要旅游城市第6位。新开通境内外航线9条，航空口岸国际直航旅客吞吐量继续保持广西第一、全国沿边地区第三的地位。

国家服务业综合改革试点区域建设扎实推进。自治区研究通过加快推进桂林国家服务业综合改革试点工作的意见。启动服务业统计、社会化养老等相关配套改革，金融创新服务平台、物流公共信息服务平台等一批示范性项目加快建设，服务业重点企业培优扶强工程全面启动，城乡商业网点建设进一步完善。第六届联合国世界旅游组织/亚太旅游协会旅游趋势与展望国际论坛、第三届中国桂林国际旅游博览会、第四届中国桂林创新创意文化节暨桂林国际动漫节成功举办，我市品牌展会影响力持续增强。开展桂林首届地方美食评选活动及各县（区）文化、旅游、商贸等节事活动。社会消费品零售总额536.35亿元，增长16%。

三、以“示范市”建设促进农业提质增效，农村发展基础进一步夯实

农业提质增效成果显著。农业“示范市”建设加快推进。农户“万元增收工程”全面实施，桂阳公路百里金桔等6条万元增收示范带初具规模；扎实开展“多播一斤种，增收百斤粮”示范工程活动，粮食总产增幅提升2.9个百分点，连续9年增产；优势特色农产品产值占农林牧渔业总产值80%以上，蔬菜、水果、肉类、水产品产量均居全区前列。林下经济产值32.68亿元，增长44.6%。高效循环农业生产模式进一步推广，自治区“无公害农产品生产示范基地县”达10个。农业产业化龙头企业年销售收入234.41亿元，直接带动13.36万农户增收。获得省级以上农业旅游示范点总数位居全区第一，“桂林休闲农业四季游”入选“中国休闲农业与乡村旅游十大精品线路”。农民人均纯收入增幅创12年来新高，全区钱粮双增现场会在我市召开，成功承办广西第三届名特优农产品交易会。

农业农村发展基础不断夯实。大力实施农村公路“畅通”“连通”工程，行政村道路通畅率提高8.2个百分点，达68.2%。完成病险水库除险加固42座、中小河流治理项目33个，新增、恢复和改善有效灌溉面积63.16万亩。建成农村饮水安全工程项目460个，解决了23.57万农村人口饮水不安全问题。实施市级以上农业科技项目34项，推广新品种300多个、新技术30多项，农业技术推广、农机耕种收一体化、农副产品购销和质量安全检测等服务体系不断完善。新农村建设扎实推进，完成400个新农村建设及1500个村屯道路硬化。扶贫工作取得新进展，全市农村贫困人口减少7.1万人，贫困村人均纯收入增长20.1%。

四、以项目投资激活增长动力，发展后劲进一步增强

重点项目建设扎实推进。实施重大项目724项，完成投资636.2亿元。湘桂铁路扩能改造工程桂林至永州段建成通车试运行，贵广铁路桂林段加紧建设；兴安至桂林高速公路基本完工，阳朔至鹿寨高速公路桂林段、灌阳（永安关）至全州（凤凰）高速公路分别累计完成总投资额的65.2%和58.8%，桂林至三江高速公路实质性开工，资源至兴安、灌阳经恭城至平乐高速公路前期工作取得重大进展。桂林市防洪及漓江补水枢纽工程川江水库大坝顺利封顶、小溶江水库大坝完成部分浇筑、斧子口水库导流隧洞全线贯通。资源金紫山风电场二期工程实现并网发电，全州天湖等4个风电场相继开工建设。

　　投资结构明显优化。实施产业类重点项目359项，完成投资占全部重点项目投资的50%以上。生产性服务业完成投资204亿元，实现跨越式增长。社会事业完成投资41.4亿元，促进了人民生活环境的改善和生活质量的提高。投资高位增长，民间投资进一步激活，民间投资达792亿元，占全社会固定资产投资的54.2%，增长37%。

五、以特色城镇建设改善城乡面貌，新型城镇化进程进一步加快

桂林世界旅游城建设全面推进。核心区新开工及续建项目67项，完成投资46.87亿元，增长42.9%。临桂大道等7条道路建成通车，新区路网逐步成型；西二环路临桂段基本具备通车条件，连接新区老城新的快速通道基本形成；水系及园林绿化工程取得阶段性成果，创业大厦、“一院两馆”、报业大厦、建设大厦等重点项目主体工程全部完成，新区整体形象进一步彰显。临苏产业带加快建设。九美桥时尚园、国际足球文化产业园等产业项目加紧实施，保利文化产业园、国奥凤凰体育休闲旅游区等项目成功引进，产城互动不断增强。

老城疏解改造提升加速。历史文化名城通过国家复核评估。桂林园博园如期竣工开园，确保了第二届广西（桂林）园林园艺博览会圆满成功，创造了园博速度和园博精神。城市重要交通节点改造提升基本完成，东安路、红岭路建成通车，福利路、阳江路等加快建设，老城交通网络逐步完善。“两江四湖”二期工程桃花江段竣工通航，南溪河清淤、截污和驳岸基本完成，城区污水集中处理率达90.5%，垃圾无害化处理率保持100%，“城乡清洁工程”进一步深化，城市环境不断改善。城市道路交通运输及户外广告管理进一步加强，城市公交覆盖范围扩大。

县城和小城镇建设步伐加快。全力实施“百镇千村”计划，完成13个镇总体规划及1280个村规划的编制，全州、平乐、灌阳等县新区建设继续推进，县城路、桥、水、电等基础设施不断完善，产业和人口集聚能力不断提高。城乡风貌改造四期工程全面完成，资源等县老城风貌改造效果明显，阳朔县兴坪镇、龙胜各族自治县龙脊村、平乐县大塘口村、秀峰区鲁家村等列入自治区第一批特色名镇（村）命名单位。

六、以生态建设提升可持续发展能力，城乡生态环境进一步改善

保护漓江力度加大。贯彻落实自治区漓江流域生态环境保护条例，编制漓江保护和开发规划，启动漓江喀斯特地貌申报“第二期中国南方喀斯特”世界自然遗产工作，猫儿山自然保护区列入联合国教科文组织“世界人与生物圈保护区网络”。漓江水源林保护、水域管理及沿岸绿化美化花化果化等工作得到加强。

生态环境建设成效显著。国家环保模范城市通过复核，在长江中下游流域污染防治工作考核中名列第一，城市环境综合整治定量考核连续17年位居全区第一，成为全区首个国家级低碳试点城市。以环境倒逼机制推动产业转型升级，规模以上工业万元增加值能耗下降12%，污染物排放总量均控制在自治区下达的控制指标范围内，全年城区空气质量优良天数达351天。“绿满八桂”造林绿化工程获全区先进，全市森林覆盖率70.94%，城市绿化覆盖率43.72%。全面实施生态市建设规划，灌阳、恭城、平乐、资源被评为国家生态文明建设工程示范县，恭城镇等15个乡镇获国家级生态乡镇称号，34个村被自治区命名为生态村。实施农村环境连片整治示范项目239个，占全区总数的三分之一。加强基本农田保护和耕地后备资源开发，连续12年实现耕地占补平衡。

七、以文化建设增强城市软实力，历史文化名城影响力进一步彰显

文化事业全面进步。在全区率先完成市县两级国有文艺院团改制，组建桂林市文艺演出有限责任公司、桂林市戏剧创作研究院（桂林市非物质文化遗产保护传承中心）。在全区率先组建新华书店有限公司，市新华书店体制改革完成。农家书屋覆盖所有行政村，文化场馆全面免费开放。“百姓大舞台”“百姓文化大讲坛”“漓江之声”等公益性文化活动品牌深受群众欢迎。廉政彩调剧《一品油茶七品官》和桂剧《月影斩》获第八届广西剧展金奖。恭城瑶族八音等10个非物质文化遗产项目列入第四批自治区级保护名录。灵渠申报世界文化遗产工作正式启动。131个村级公共服务中心全部竣工。提前完成20户以下广播电视“村村通”工程，受益用户10万多户。

文化产业加快发展。实施文化发展规划和文化产业发展规划。高新区创意产业园等4家企业被命名为第四批自治区级文化产业示范基地，兴安、阳朔被命名为首批自治区特色文化产业示范县，临桂、龙胜、恭城被命名为首批自治区特色文化产业项目示范县。以演艺娱乐、游戏动漫、出版发行、印刷包装等为引领的文化产业产值较快增长。

八、以体制机制创新增强经济社会发展活力，改革开放进一步深化

重点领域改革成效显著。统筹推进“扩权强县”、城乡综合配套改革试点工作，圆满完成上级审批下放事项接收和市级审批事项下放，有力推动了县域经济发展，县域地区生产总值、财政收入占全市的比重分别达70.9%和50.7%，一批经济发展较快的县进入广西科学发展10佳县、进步县行列。统计方法制度改革有效推进，“四大工程”建设取得实质性成果。国企改革取得新进展，内燃机配件厂、矿山机械厂等困难企业成功改制重组。集体林权制度主体改革全面完成，林地流转试点顺利实施。医药卫生体制改革不断深化，永福、兴安县公立医院改革试点为全区提供了成功经验，医患纠纷调解新机制和工作模式在全区推广。全民医保体系进一步健全，新农合筹资标准和财政补助标准提高。积极实施事业单位绩效工资，企业职工工资正常增长和支付保障机制不断完善。加强金融体系和投融资平台建设，新增金融机构和新型金融组织15家。支持民营经济加快发展，规模以上民营企业超500家。

创新能力持续提升。培育建设自治区级工程院5家、研发中心25家、企业技术中心6家，32项科技成果获自治区表彰奖励。每万人拥有发明专利1.71件，居全区首位；专利授权1252件，排全区第二位。积极实施质量兴市战略，燕京啤酒（桂林漓泉）公司获首届自治区主席质量奖。

开放合作领域全面拓展。积极参与区域合作，成功引进华能集团等一批央企及国内500强企业。在第九届中国—东盟博览会上签约项目数和投资额均列全区前茅。新签市外境内项目415个，总投资486.47亿元，增长4.3%；实施市外境内本年度到位资金项目812个，到位资金542.18亿元，创历史最好水平。对外贸易平稳发展，进出口总额保持增长。

九、以人为本推进民生改善，社会事业进一步发展

民生保障水平有了新提高。各级财政投入民生保障资金186.78亿元，增长9.5%。筹集资金66.35亿元，完成十项惠民工程。筹集资金1700多万元，对低收入居民及清真牛肉供应实行临时价格补贴，实施临时价格干预。开发公益性岗位近6000个，城镇新增就业8.5万人，城镇登记失业率3.9%。新型农村社会养老和城镇居民养老保险比全国提前半年实现制度全覆盖。继续提高城乡低保对象人均补助标准，累计发放低保金4.81亿元、城乡医疗救助金8122万元。建设保障性住房18270套，其中竣工7570套，城镇低收入住房困难家庭廉租住房租赁补贴实现应保尽保，完成农村危房和茅草树皮房改造20356户。

社会事业有了新发展。学前教育三年行动计划和深化职业教育攻坚五年计划深入实施，启动农村义务教育阶段学生营养改善计划试点，九年义务教育巩固率达95%，高校各项建设成效显著。防治艾滋病攻坚工程深入实施，出生缺陷综合防治工作经验在全国推广。参加自治区首届全民健身运动会、第七届农民运动会取得优异成绩，成功承办和举办桂林国际半程马拉松赛和桂林市第二届少数民族传统体育运动会等体育赛事。诚信计生提质扩面持续深入，我市首创的诚信计生模式已上升为地方性法规，在全区推广。

社会管理有了新进步。社会管理创新综合试点、社会管理信息化建设顺利推进。城乡社区服务组织和基层管理服务体系进一步完善，矛盾纠纷排查、调处、化解工作及群众维权机制不断健全，信访维稳工作取得新成效。全面开展“六五”普法，加强平安桂林建设，构建社会治安防控体系，严厉打击各类犯罪活动，进一步加强环境、道路交通、消防、生产、食品药品等重点领域安全管理及应急管理，人民群众安全感和满意度进一步提升。

一年来，我们切实转变政府职能，坚持科学决策、民主决策，坚持依法行政、勤政廉政，切实推进政务公开和政府信息公开，切实推进党风廉政建设，加强行政监察和审计监督，推进政风行风建设和绩效管理；坚持重大事项向市人大及其常委会报告、向市政协通报制度，办理人大代表建议58件、政协提案270件，办结率100%；广泛听取各民主党派、工商联、无党派人士意见，支持工会、共青团、妇联等群团组织发挥作用。国防教育、国防后备力量建设、“双拥”工作得到加强。民族、宗教、外事、侨务、接待、编制、档案、保密、机关事务、气象、测绘、防震减灾、发展研究、地方志、社会科学、老龄、残疾人等事业取得新进步，港澳工作、对台工作取得新进展。中直、区直驻桂林单位取得新成绩。

一年来，我市经济社会发展在全国、全区取得了多项荣誉。荣获“全国‘两基’工作先进地区”“全国优秀创业先进型城市“全国未成年人思想道德工作建设先进城市”“全国对口支援三峡工程库区移民工作先进集体”“2011—2012年度中国十大魅力会展城市”等荣誉称号，获“全区第八届环境综合整治‘南珠杯’特等奖“广西人口和计划生育工作特别先进奖“广西住房保障工作先进集体”等7项自治区级荣誉。市直各部门、各县（区）共荣获国家级荣誉48项，自治区级荣誉超过100项。这些成绩的取得，是市委正确领导、市人大、市政协监督支持及全市人民团结奋斗的结果。在此，我代表市人民政府，向全市各族人民，向驻我市中央、自治区单位、人民解放军和武警部队官兵，向各民主党派、工商联、人民团体以及海内外所有关心支持和帮助桂林发展的各界人士，致以崇高的敬意和衷心的感谢！

在肯定成绩的同时，我们也清醒地认识到，我市在发展中还面临不少困难和挑战，政府工作也还存在着一些差距和不足：整体经济实力不够强，还需要在提高总量、提升质量上狠下功夫；城市经济辐射带动力不足，还需要在统筹城乡协调发展上狠下功夫；大企业、大项目、大园区不够多，还需要在优化环境、招大引强上狠下功夫；城市管理存在薄弱环节，还需要在完善城市功能、提高管理水平上狠下功夫。对此，我们将高度重视，认真研究，务实解决。

2013年工作总体要求和目标任务

今年是全面贯彻落实党的十八大精神的开局之年，是全面建设桂林国际旅游胜地的起步之年。我市正处于加快富民强市新跨越的黄金机遇期和打造桂林国际旅游胜地、建设美丽桂林、与全国同步建成小康社会的关键时期。经过多年努力，我市的发展优势日益凸显，城市综合竞争力和持续发展能力明显增强。我们既要充分认识发展形势的积极方面，坚定发展信心，又要运用“底线思维”，充分估计发展中面临的困难和挑战，认真谋划全局，增强应对能力，牢牢把握发展主动权。

2013年政府工作的总体要求是：全面贯彻党的十八大和中央经济工作会议精神，按照自治区党委、政府和市委的工作部署，坚持以邓小平理论、“三个代表”重要思想、科学发展观为指导，坚持走农业稳市、文化立市、旅游兴市、工业强市之路，牢牢把握主题主线，以提高经济增长质量和效益为中心，以“三个率先”为目标，以“三个全面”为重点，按照“加快建设新城，着力提升老城，城乡协调推进，产业融合发展，生态文化结合，富裕和谐桂林”的部署要求，努力增强发展的动力和活力，实现建设桂林国际旅游胜地、建设美丽桂林良好开局，为加快富民强市新跨越、与全国同步全面建成小康社会打下坚实基础。

今年经济社会发展主要预期目标是：地区生产总值增长11%，财政收入增长12%，全社会固定资产投资增长20%，规模以上工业增加值增长18%。社会消费品零售总额增长16%，外贸进出口总额增长10%。万元地区生产总值能耗下降3%，氮氧化物排放量削减5.618%，二氧化硫、化学需氧量、氨氮排放量均控制在自治区许可范围内。城镇居民人均可支配收入增长13%，农民人均纯收入增长14%，居民消费价格涨幅控制在4%左右。城镇登记失业率控制在4.5%以内，人口自然增长率控制在9‰以内。

2013年工作部署

为实现今年的工作目标任务，要重点抓好以下工作：

一、全面推进桂林国际旅游胜地建设，提升发展现代服务业

围绕《桂林国际旅游胜地建设发展规划纲要》，按照世界水准、国际一流、国内领先要求，以创新体制机制为动力，以政策支持和重大项目建设为支撑，以推进旅游产业转型升级为重点，确保国际旅游胜地建设良好开局。力争旅游总人数增长6%，旅游总收入增长25%；入境游客人数增长5%，旅游外汇收入增长10%。服务业增加值增长10%。

提升桂林旅游发展定位。把握建设世界一流旅游目的地、全国生态文明建设示范区、全国旅游创新发展先行区、区域性文化旅游中心和国际交流重要平台的战略定位，按照国际化要求及先进管理理念，以突出旅游功能、提升生态功能、增强城市功能为导向，高起点、高标准编制国际旅游胜地建设相关配套规划；提升旅游综合服务功能，强化旅游发展要素保障，培育旅游新业态，创新旅游业与其他产业融合发展新模式，加快形成服务经济为主导、特色经济为支撑的现代产业结构；探索以旅游为纽带的城乡统筹协调发展新途径，加快形成城市空间布局、产业布局、基础设施、城市建设和管理、生态环保、文化教育等与国际旅游胜地建设协调统一的整体发展格局。

加快旅游项目建设。围绕推进《规划纲要》提出的“六个一批”重点工程，全面加快旅游产业及配套重大项目建设。实施旅游胜地重大项目584项，年度完成投资566亿元。以建设桂林世界旅游城为突破口，开工并加快建设保利文化产业园、国奥凤凰体育休闲旅游区、国际足球文化产业园、九美桥时尚园等项目，加大旅游综合体、旅游特色街区等旅游产业项目招商引资力度。科学合理布局，建设提升旅游景区景点、旅游基础设施、旅游服务基地、休闲养生度假酒店，加快实施城北体育文化城、地中海俱乐部桂林度假村、桃花江旅游休闲度假区及特色旅游休闲街区等中心城区文化休闲度假旅游项目，启动正阳路东巷保护整治提升工程，提升中心城区旅游功能；加强重点旅游县建设，着力打造特色旅游镇村品牌，统筹推进县域重大旅游项目建设。积极争取政策支持，组织专门班子，配备专门力量，全面梳理《规划纲要》涉及的具体政策和项目，加强与国家、自治区有关部门沟通衔接，力争国家和自治区支持旅游胜地建设的政策尽早落地实施，争取更多的项目和资金支持。

实施“品质旅游”战略。着力打造旅游消费层次丰富、产品体系完整、产品线路完善、服务水平一流、市场规范的旅游精品和旅游品牌。整合全市优势资源，丰富提升山水观光、休闲度假、历史文化、生态乡村、民族文化演艺、红色旅游、户外运动、浪漫婚典等八大系列旅游产品。加大旅游整体国际形象宣传推介和旅游促销力度。办好第七届联合国世界旅游组织/亚太旅游协会旅游趋势与展望国际论坛、第四届桂林国际山水文化旅游节、第四届中国桂林国际旅游博览会等品牌旅游展会。积极发展智慧旅游，打造一流旅游电子商务平台，提高旅游信息化、国际化水平。开展“国际旅游胜地美丽桂林休闲游”主题年整体宣传促销活动，培植稳定的消费群体，积极发展高端旅游，深入拓展国际及港澳台入境旅游市场；继续实施旅游航线包机奖励政策，开发培育航线和旅游包机。推进旅游管理体制机制创新，加强旅游公共服务体系建设，完善漓江5A级景区长效管理机制，加强旅游标准化、旅游安全保障、旅游市场监管和旅游诚信体系建设，进一步规范旅游市场秩序；支持旅游企业转型升级、做大做强，开展旅游服务质量系列考核评比活动。加强旅游区域合作，进一步强化与珠三角、长三角、西南、港澳台等区域城市的旅游交流合作，充分利用中国—东盟博览会平台，加强与东盟国家旅游合作。大力发展旅游职业教育，打造国际旅游教育基地。建立健全工作体制机制，制定实施近期行动计划，引入国内外智力支持，开展国际旅游胜地专项研究，强化空间资源优化利用及产业技术、人才智力支撑，保障国际旅游胜地建设强力推进。

促进相关服务业与旅游业融合发展。全面落实自治区加快推进桂林国家服务业综合改革试点工作的意见。加快发展“1个核心区、2个功能集聚带、5大示范区”，加速构建服务业“345”产业体系。建立服务业引导基金，加大对服务业集聚区、重点项目和企业的扶持，加强试点领域标准体系和品牌建设。积极推进国际卫生空港建设，提升出入境能力。推进乡村生态休闲旅游、服务业信息平台、旅游与会展资源整合及产业协同等国家级服务业试点示范项目建设。运用政府引导、市场运作等综合手段，加大品牌展会引进力度，筹办养老产业国际论坛，加快打造国际会展名城。对服务业重点企业给予政策扶持，促进服务业转型升级。加快建立扩大消费需求长效机制，积极培育汽车、健康、养生、家政服务等消费热点，加快完善城乡商贸流通体系，启动临桂新区商贸物流园、桂林西货运枢纽及物流基地规划建设，继续实施“万村千乡市场工程”。实施“电子商务进社区”试点建设，申报国家级电子商务示范基地。全面启动再生资源回收体系建设，积极创建全国再生资源回收利用试点城市。严格落实房地产调控政策，促进房地产市场健康发展。

二、推进产业转型升级，加快工业和信息化发展

实现富民强市的宏伟目标，关键在于发展壮大产业、做大做强工业。要走高端、低碳、生态、环保的特色产业发展道路，大力推进工业升级改造，着力培育发展大园区、大产业、大项目、大企业，着力发展特色效益工业，打造工业发展新优势。力争全部工业总产值超2000亿元，达2150亿元，增长18%；规模以上工业总产值1900亿元、增加值580亿元，分别增长20%和18%。

持续推进产业结构调整优化。完成工业投资640亿元，增长20%；技术改造投资539亿元，增长30%。开工建设亚科新材料生产基地等30个投资超亿元项目，续建三金药业现代中药产业化基地等50个投资超亿元项目，重点推进激光所高清视音频传输系统及产业化等15个投资超亿元项目前期工作，着力推动600项投资500万元以上的技术改造项目。以重点项目建设为突破口，加快推动电子信息、生物医药、新能源及汽车等重点产业和新兴产业建设，完善工业配套，引导新兴产业集聚发展，力争新兴产业产值突破450亿元，增长25%。

强力推进千亿元园区和千亿元产业建设。高新区以打造千亿元创新型特色园区为目标，在拓展空间，培育优势，完善配套，创新驱动，改善政务环境上下功夫，力争技工贸总收入突破700亿元。扎实推进高新区新一代信息技术和创意动漫产业园、苏桥经济开发区汽车产业园、西城经济开发区先进装备制造产业园、雁山低碳经济产业示范园等特色产业园区建设。积极实施“飞地工业”新模式，拓展老城区产业发展空间，启动宝山工业园建设，加快发展临苏产业带。坚持“产城一体”发展模式，加快园区综合服务配套设施建设，推进园区与城镇化互动。力争工业产值突破50亿元园区达到3个、100亿元园区达到4个，西城经济开发区工业产值突破300亿元。继续支持以光伏、风能为重点的新能源产业发展，努力打造千亿元产业。

深入推进“抓大壮小扶微”工程。积极引进国内外行业龙头企业，加快培育一批主业突出、创新力强、关联度大、带动性强的骨干企业。加强中小企业服务平台和创业基地建设，强化融资服务，筹建企业应急互助资金，支持实体经济发展，推动中小企业上规模、上台阶。力争新增10亿元以上企业2家、亿元以上企业30家、规模以上企业60家，新增各类微型企业2500家。

全面推进信息化建设。实施中小企业网上营销工程，着力发展以云计算、物联网、移动通信为代表的新一代信息技术，推动信息化与工业化深度融合。大力推进“智慧城市”和“宽带桂林”建设，搭建云计算公共服务、地理信息共享和视频资源管理三大平台。强化信息安全保障。

三、突出发展现代农业，促进农业大市向强市转变

深入实施“四大流域”和西部山区现代农业发展规划，提升特色农业发展水平。力争完成农林牧渔总产值438亿元，增长5.8%，增加值增长5.3%。

加快打造现代农业产业体系。巩固提升6条农业“万元增收”示范带建设，做大做强特色效益农业，实现粮食、水果、蔬菜、食用菌、毛竹、花卉苗木、生猪、家禽等优势特色农产品产值持续增长。重点规划建设以雁山区雁山镇、临桂县会仙镇为核心的农业科技示范园区；建设灵川大圩、临桂两江、阳朔白沙等10个水肥一体化示范基地；建设全州稻灯鱼菇、兴安一年两收葡萄、恭城休闲农业等10个富民增收万元片区。推进建设一批规模养殖场、养殖小区，推广标准化、设施化、工厂化生态养殖。大力发展特色林下经济，实现产值36亿元。深入拓展循环农业模式，加大农业良种良法推广力度，大力发展新型农业耕作模式，提升“三避”栽培、测土配方施肥等先进实用技术。推进农业向接二产连三产延伸，打造一批休闲农业精品，促进休闲农业提档升级。全力打造全国、全区农产品知名品牌，重点优化提升6大种植业特色优势农产品基地。加快规划建设区域性农产品加工物流园区，不断完善农产品专业批发市场及冷藏、物流设施。推进农业生产经营体制改革，大力培育农业生产经营主体，提高农业组织化程度和专业化水平。大力发展农产品采后处理、贮运加工产业，积极推动农超、农校、农批、农企等产销对接。

加强农业农村基础设施建设。实施153座中小型病险水库除险加固工程，抓好桂江、湘江等重要支流及中小河流治理，推进兴安、荔浦等中央小型农田水利项目县建设，加快青狮潭和峻山大型灌区节水改造，继续实施农村饮水安全项目，解决25万农村人口饮水不安全问题。加快实施“通畅”和“连通”工程，新增通自然村（屯）道路200公里以上，130个建制村通水泥路，通畅率达76%。推进农村水电增效扩容和电网升级改造工程。加强农机装备建设，力争农业耕种收综合机械化率达40.5%。深入推进社会主义新农村建设，大力推广阳朔田园风光休闲型、恭城生态农业带动型、桂北民俗风情体验型等建设发展模式，建成“普惠制”新农村400个。继续实施城乡风貌改造工程。

全力保障农产品安全有效供给。保持粮食播种面积563万亩、总产198万吨以上。健全“菜篮子”供给体系，突出抓好以临桂、灵川、雁山为重点的城郊“菜篮子”蔬菜种植基地和县域“菜篮子”基地建设。强化农产品生产、流通和消费安全监管，抓好农产品标准化生产基地建设，推进无公害农产品、绿色食品、有机农产品和农产品地理标志认证服务和监督管理，完善动植物疫病防控和农产品质量安全追溯监控体系，确保农产品从田间到餐桌的质量安全。

四、拓展优化城镇格局，推动城乡一体化发展

坚持规划要硬、机制要活、管理要细、工作要实的理念，强化规划统一管理，突出桂林地方特点，打造桂林城市特色。抓住行政区划调整有利时机，全面加快桂林世界旅游城、各县县城以及特色乡镇建设，推进区域协调、城乡互动、产城融合，在城乡统筹、城乡一体化发展上有新作为，力争城镇化率达44%。

加快建设桂林世界旅游城。深入实施“保护漓江，发展临桂，再造一个新桂林”战略，优化、完善、提升世界旅游城规划，加快基础设施、产业、民生等重点项目建设，实施世界旅游城项目252项，完成投资186亿元以上。围绕提升新区整体形象，加快世界旅游城核心区项目建设，力争新开工16项、续建57项、完工13项，完成投资58亿元以上。确保创业大厦、“一院两馆”、报业大厦、建设大厦等标志性项目年内建成使用；加快西城大道（提升改造）等8条道路建设，年内实现新区结构性干道全线贯通；旅游景观水系主体工程基本完成；开工建设新雁路、新区综合医院、桂林中学新校区以及高端休闲街区、休闲商务酒店、市政和生活配套设施、街道及社区服务设施等一批新的重大项目，积极推动教育卫生、文化体育、旅游休闲、商住娱乐等优势资源向新区转移。全面启动新区城市管理工作，提升社区社会服务功能。加快世界旅游城三大旅游功能板块建设发展，大力推进凤凰体育运动休闲旅游区、万福休闲度假旅游区、以会仙喀斯特国家湿地公园为重点的会仙湿地旅游区建设。统筹新区老城资源，引入战略投资，规划建设一批大型城市综合体。

强力疏解改造提升老城。全面实施城市交通畅通优化工程，加大老城路桥基础设施建设力度。实施项目24项，完成投资35.76亿元。基本完成西二环路、滨江北路、芳香路等项目建设；完成福利路一期、站前路、芳华路等项目建设；新开工建设万福东路、龙门大桥、阳江北路等项目；开展琴潭南路等项目前期工作，进一步完善纵贯南北、连通东西、衔接老城新区的交通网络。完善老城功能，加强重要节点及公共服务设施改造建设；注重商业业态的统筹谋划，加快站前路商业广场等产业项目建设；推进城乡结合部和城中村改造，切实改造一片、提升一片。加快城市水系及周边环境综合治理和风貌街区改造，进一步彰显生态山水名市和历史文化名城特质。

着力提高城市管理水平。以建设国际旅游胜地为标准，以提升老城品质为目标，着力完善城市功能，规划、建设、改造提升一批专业市场和农贸市场；推行公交优先政策，充分利用现有空间，引进先进技术设施和管理手段，解决中心城区停车难问题；坚持疏堵结合，加大整治力度，组织各级机关和企事业单位带头履行门前三包责任，动员全社会力量深入实施“城乡清洁工程”，认真清理卫生死角，开展违法建筑、占道经营、交通秩序等系列专项整治，建立长效管理机制。多渠道汇集社会资金，加大城市管理投入，切实加强城市规划、建设和管理。实施城市管理教育培训工程，学习借鉴境内外城市管理先进经验。加强舆论监督和社会监督，实行严格的定责、考责、问责制度，确保城市管理取得广大市民和游客看得见、感觉得到、普遍满意的实效。

大力优化城镇结构。整合县域资源，注重科学布局，积极培育中等城市，促进大中小城市协调发展，全力构建以中心城为重点，县城新区和重点镇为支撑，大小结合、功能互补、特色鲜明的城镇群和城镇带。加快各县县城和重点特色乡镇建设，扩大规模、完善基础设施，提升综合承载能力和服务能力。积极争取区划调整，力促县城所在地逐步撤镇改街道，积极推动撤乡改镇。

五、强化项目建设支撑，提升投资对经济增长的拉动力

进一步优化投资结构，保持投资合理较快增长，统筹推进重大项目建设。全年计划实施重点项目690项，完成投资640亿元。

加快交通、水利、能源重大基础设施建设。打造区域性综合交通运输枢纽和国家公路运输枢纽城市，完成交通建设投资105亿元，推进贵广铁路桂林段建设；加快建设桂林至三江、阳朔至鹿寨、灌阳（永安关）至全州（凤凰）高速公路，开工建设资源至兴安高速公路，力争自治区尽快开工建设荔浦至玉林高速公路，加快灌阳经恭城至平乐高速公路前期工作和招商引资；争取桂林两江国际机场航站楼及站坪配套设施扩建工程开工建设。完成水利项目建设投资26亿元，力争小溶江水库完成大坝封顶，斧子口水库实现大江截流；全面启动桂林市漓江堤岸修复工程等重大水利项目的前期工作。完成能源建设投资41.7亿元，实现兴安源江等3个风电场竣工，加快全州天湖风电场一期、恭城燕子山风电场建设，开工建设兴安平岭、恭城西岭、全州六字界等6个风电场，使我市风力发电装机容量达到65万千瓦。

继续优化投资结构。准确把握国家产业政策、资金投向，围绕国际旅游胜地、优势产业及战略性新兴产业、基础设施、“三农”、社会事业和社会管理、节能减排和生态建设等重点领域，谋划和储备重大项目，加大投资力度。把拓宽资金来源渠道作为确保投资增长的主攻点，积极发挥财政资金的导向作用，加大财政项目资金及政府债务的管控力度；加强金融对实体经济的支撑，密切政银企合作，充分发挥政府融资平台作用；破除制约民间投资的“弹簧门”“玻璃门”，切实降低民营投资准入门槛，扩大民营投资准入领域，实现投资主体多元化。力争新增贷款157亿元以上，全社会融资总量1650亿元，争取国家核准发行企业债券10亿元。

全力抓好项目服务。优化投资环境，提高工作效率，加快项目前期和项目审批工作进度，加强项目管理和协调服务。抓好旅游产业用地改革、城镇低效用地再开发试点工作，统筹运用“区位调整”“增减挂钩”“只征不转”“征转分离”等政策措施，努力保障项目建设用地，着力解决征地搬迁安置等“瓶颈”问题。

六、全面启动城乡居民收入倍增计划，切实提高群众生活水平

促进居民收入增长与经济发展同步，为城乡居民收入率先达到全国平均水平打好基础。突出农民、企业职工、中低收入群体增收，力争农民人均纯收入达8300元以上、城镇居民人均可支配收入达25000元以上。

实施农民收入倍增计划。加快发展特色效益农业，大力实施农户“万元增收工程”“钱粮双增工程”和“百村示范、千村推进”工程，推进“吨粮万元田”“万元片区”“万元增收村屯”和“10万元致富示范户”建设，提高农民家庭经营性收入。促进农民就业创业，深入实施百万农民就业培训工程，推动农村富余劳动力向城镇和非农产业有序转移，大力扶持农民回乡创业，农村劳动力转移就业新增8.5万人，增加农民工资性收入。强化政策支持，加大财政、金融、科技、保险等支农力度，落实各项生产补贴政策，新型农村养老保险基金人均每月增加20元，农村低保对象补助人均每月增加10元，新农合筹资标准由290元提高到340元，完善农村“一事一议”筹资筹劳办法，增加农民转移性收入。完善农村土地、林权等产权制度，发展适度规模经营，逐步培育发展家庭农场，壮大农村集体经济，提高农民财产性收入。坚持专项扶贫、行业扶贫、社会扶贫共同推进，加快实施整村推进扶贫开发，重点抓好产业扶贫工程，力争减少贫困人口7万人以上，贫困农民人均收入增幅高于全市农民人均收入增幅。

实施城镇居民收入倍增计划。加快完善企业工资正常增长机制，建立健全工资集体协商和工资保证金制度，促进劳动者工资水平与企业经济效益有效衔接。完善事业单位收入增长机制，加快实施事业单位绩效工资，促进事业单位工资合理有序增长。提高城镇社会保障水平，建立健全城镇居民社保待遇正常增长机制，医保补贴标准由每人每年240元提高到280元，低保对象补助人均每月增加20元，进一步完善低收入居民临时价格补贴联动机制，加快推进平价商店建设试点。促进全民创业，扎实推进创业型城市建设，全面落实促进民营经济健康发展的各项政策，制定完善配套措施，扶持民营经济发展。抓好就业工作，积极创造城镇就业岗位，加大对城镇困难群体就业创业帮扶力度，城镇新增就业6.4万人，确保“零就业家庭”至少有1人就业。

七、深入实施文化立市战略，促进文化大发展大繁荣

把推动文化大发展大繁荣作为桂林国际旅游胜地建设的战略之举，加快实施文化建设“五大工程”，兴起文化建设新高潮，提升城市软实力。

深入推进文化精神工程。加强社会主义核心价值体系建设，大力弘扬民族精神和时代精神，积极倡导和践行广西精神，培养塑造城市人文精神。广泛开展社会主义核心价值观进机关、进学校、进企业、进村屯、进社区学习教育活动。深入推进全国文明城市和群众性精神文明创建活动，切实提高公民思想道德素质，培育良好社会风尚。

深入推进文化景观工程。加快靖江王府及王陵、甑皮岩国家考古遗址公园建设。继续做好兴安灵渠申报世界文化遗产工作。推进特色文化街区建设。

深入推进文化精品工程。加强文化产品创作生产，打造人民群众喜闻乐见、具有桂林特色的文学、美术、戏曲、民族歌舞等优秀作品。提升“百姓大舞台”“百姓文化大讲坛”“漓江之声”“周末大家乐”等群众文化品牌。围绕“演艺之都、创意福地、富集市场、智慧新城”定位，重点培育发展文化创意、演艺娱乐、动漫游戏、艺术品及艺术收藏、出版印刷等产业，推动文化与相关产业融合发展。努力扩大中国桂林创新创意文化节暨桂林动漫节等精品文化会展的影响力。支持广西师范大学出版社、漓江出版社做大做强，打造广西数字出版的重要基地。

深入推进文化惠民工程。抓好村级公共服务中心建设，推进临桂国家公共文化服务体系示范项目申报工作。继续深化公共图书馆、博物馆、文化馆免费开放，加快推动已建成的1684个农家书屋向以用为主转变。努力实现广播电视从“村村通”到“户户通”“长期通”，做好农村公益电影放映服务。统筹文化节庆活动，构建桂林特色文化节庆体系，推动基层群众文化活动蓬勃开展。

深入推进文化管理工程。继续推进文化体制改革，加强培育现代文化市场体系。开展文化市场专项治理，深入开展“扫黄打非”，净化文化市场。加强传统风貌、文物及非物质文化遗产保护和利用。

八、加强生态文明建设，努力建设美丽桂林

牢固树立尊重自然、顺应自然、保护自然的生态文明理念，率先走出一条在保护中加快发展、在发展中加强保护的路子，建设美丽桂林，实现永续发展。

加强漓江科学保护。全面实施漓江流域生态环境保护条例，大力推进科学保护漓江“六大工程”，启动漓江保护科技工程。加强漓江喀斯特地貌申报世界自然遗产的相关工作，完善漓江生态补偿机制，提高水源林生态功能，提升绿化美化水平。加强漓江航道及主要支流河道整治，严格保护洲岛景观，强化沿岸石山及洞穴保护开发与安全管理。推进沿岸村镇规划管理。

加强环境保护和节能减排。实施农村环境连片整治，继续推进生态市、生态县、生态乡镇、生态村建设。积极开展国家森林城市创建活动，全面推进“绿满八桂”造林绿化工程，提高全市森林覆盖率和城市绿化覆盖率。优化国土空间开发布局，落实最严格的耕地保护制度，推进土地开发复垦整理、农村土地综合整治，鼓励农村土地有序流转，积极创建节约集约用地模范县（区）。加强污染防治，防止重金属污染、饮用水源污染等突出环境问题发生；开展青狮潭水库环境专项治理，继续加强城市和村镇垃圾收集处理设施建设，因地制宜推进县域生活垃圾“村收镇运县处理”，探索边远乡村生产生活垃圾处理新模式，加大城镇生活污水处理配套管网、园区及企业治污设施等环保项目建设和管理力度，确保环境安全。鼓励发展节能、降耗、减污的高新技术产业，加大淘汰落后产能力度，培育工业循环经济示范企业和园区，大力发展生态农业和有机农业。加快建设低碳城市，推广应用低碳技术及产品，支持低碳旅游，推进车船使用天然气和可再生能源建筑应用城市示范工作。开展全民节能减排，加强公共机构节能。完善生态环境监管体制，强化环境监测和执法。加强市区大气环境综合整治，做好大气PM2.5实时监测、实时发布工作。

九、保障民生创新管理，强力推进和谐社会建设

以保障和改善民生为重点，加快健全基本公共服务体系，发展社会事业，实施惠民工程，创新社会管理，努力让人民群众过上更好的生活。

提高城乡社会保障水平。扎实建设覆盖城乡居民的社会保障体系。深入推进新型农村社会养老保险和城乡居民基本养老保险，继续抓好职工基本养老、失业、基本医疗、工伤、生育五项保险参保和基金征缴。进一步提高城乡低保、农村五保供养、医疗救助、孤儿救助、自然灾害和临时困难救助水平和保障标准，完善社会救助体系。抓好市残疾人康复中心、托养中心项目前期工作，落实残疾人救济抚养计划。支持发展慈善事业。建设保障性住房、棚户区改造住房8634套，分配入住5600套，实现城镇低收入家庭廉租住房补贴应保尽保，实施农村危房改造1.8万户。

坚持优先发展教育。全面实施素质教育，深化教育领域综合改革，着力提高教育质量。办好学前教育，均衡发展九年义务教育，基本普及高中阶段教育，力争全市学前教育毛入园率达76%以上，九年义务教育巩固率达95%以上，高中阶段毛入学率达87%以上。继续实施职业教育攻坚五年计划。加大教育投入，合理配置教育资源，重点向农村、边远、贫困、民族地区倾斜，支持特殊教育，促进教育公平。启动市委党校改扩建项目。积极支持高校建设。

加快卫生事业发展。巩固发展医药卫生体制改革成果，加强基本医疗保障制度、基本公共卫生服务、基层卫生基本药物制度建设，全面推进县级公立医院改革，完善新型农村合作医疗，切实抓好国家公共卫生政策的落实。进一步科学配置卫生资源，加强卫生基础设施建设，完善区域卫生信息平台。提高疾病防控能力，继续推进防治艾滋病攻坚工程。着力打造文化卫生、品牌卫生、放心卫生。

积极发展体育事业。广泛开展全民健身运动，促进群众体育和竞技体育全面发展。继续举办全市大众篮球赛、“五人制”足球赛等赛事。注重体育与旅游、文化相结合，加快体育旅游基础设施以及高端运动休闲项目建设。

　　加强人口和计划生育工作。继续抓好诚信计生的巩固提升扩面工作。强化计生优质服务，提高出生人口素质。促进各项民生政策与人口计生利益导向政策的衔接落实。

创新和加强社会管理。按照“党委领导、政府负责、社会协同、公众参与、法制保障”的要求，加强基层社会管理和服务体系建设，增强城乡社区服务功能，发挥群众参与社会管理的基础作用。积极推进户籍制度改革，加强流动人口、农村留守人员和特殊人群的管理服务。深入开展“六五”普法。扎实开展社会矛盾排查化解工作，畅通和规范群众诉求表达、利益协调、权益保障渠道。深入推进平安建设和法治建设，加强新形势下社会治安防控体系建设，强化司法基本保障，依法防范和惩治违法犯罪活动，保障人民生命财产安全。切实加强安全生产、食品药品安全、环境安全以及应急管理等工作，增强人民群众安全感和满意度，确保社会和谐稳定。深入开展国防教育，扎实做好国防动员、民兵预备役、人民防空和“双拥”工作，做好退役军人就业工作。

实施为民办实事工程。筹措资金37亿元以上，完成10项惠民工程。（1）社保惠民工程。实施城乡居民社会养老保险、城乡最低生活保障、五保村建设、阳光家园计划、贫困残疾人家庭无障碍改造等项目。（2）健康惠民工程。实施新型农村合作医疗、地中海贫血防治、城区社会卫生服务机构建设、免费孕前优生健康检查等项目。（3）教育惠民工程。实施职业教育基础能力建设、扩大农村学前教育资源、农村义务教育学生营养改善计划、学生资助等项目。（4）强基惠民工程。实施凯风路（第八中学至电厂铁路专用线段）内涝治理工程、修建贫困村屯道路、小型农田水利重点县建设、农村安全饮水解困工程、“普惠制”新村建设、自然村屯内道路硬化、城区新农村建设、整村推进扶贫开发等项目。（5）安居惠民工程。实施农村危房改造、新建公共租赁住房、新建廉租住房、城市棚户区改造等项目。（6）土地整治惠民工程。按照项目计划实施好土地整治、农业综合开发土地治理等项目。（7）农补惠民工程。实施农补惠民、农资综合补贴、粮食作物良种补贴等项目。（8）生态惠民工程。实施青狮潭西干渠临桂县城段综合美化治理、“绿满八桂”造林绿化、石漠化综合治理、森林生态效益补偿、农村环境连片整治示范、太阳能路灯推广等项目。（9）文化惠民工程。实施村级公共服务中心建设、农民体育健身工程等项目。（10）通畅惠民工程。实施LED路灯节能改造、公交优先便民、建制村建设沥青水泥路等项目。

十、深化改革扩大开放，打造创新发展新优势

深化重点领域和关键环节改革。推进城乡综合改革，激活农村生产要素，改善农村生产方式和农民生活方式，推进城乡规划一体化、公共服务均等化、经济社会权利平等化，在坚持家庭承包经营制度的基础上，引导城市工商资本投入农业农村。继续深化国有企业改革，推进国有资产管理体制创新。引入市场机制，推动市政、市容、园林等公用事业改革。大力发展金融市场，鼓励金融机构创新金融产品和服务方式，大力支持企业上市，培育一批高成长性的上市后备企业。抓好第三次全国经济普查，推进城乡居民收入一体化住户调查、服务业统计改革试点和平台建设。

提高开放合作水平。加大“央企入桂”“名企入桂”工作力度，积极推动产业招商、项目招商、以商招商、区域招商，继续做好“大兑现”工作，掀起新一轮招商引资高潮，力争实际利用外资和市外资金分别比上年任务增长10%以上。大力推进区域战略合作，继续深化与周边地区和发达地区的合作交流，发挥产业互补优势，谋求共赢发展。加快“走出去”步伐，支持企业参与境外资源开发和经济合作。稳定发展对外贸易，实现进出口总额10.73亿美元，增长10%。

创新城区经济发展方式。进一步完善市、区财政管理体制，简化行政审批环节，建立责权利相统一的管理运行机制，增强城区经济发展动力与活力。进一步明确城区功能定位，总体布局城区产业发展，建立差异化的城区考核机制，鼓励城区依托自身优势，培育产业集群，打造产业品牌，促进形成各具特色、合作互补、共赢共荣的城区经济发展格局。创新城区经济载体建设，促进城区产业转型升级，力求城区在高新技术产业、旅游商贸物流业及生态休闲观光农业等重点产业发展上取得突破。

大力实施创新驱动战略。加快建立产业优化、园区建设、人才引进、要素聚集、成果转化等为重点的区域创新体系，加快建立以企业为主体、市场为导向、产学研用相结合的科技创新平台，积极发挥大中专院校及科研院所作用，建设新兴产业研发“孵化”、多元化科技创新公共服务平台，培育建设国家级工程技术研究中心和企业技术中心，加强自治区级工程院、研发中心、工程技术研究中心建设，提高产业核心竞争力，打造一批具有核心竞争力的骨干企业，培育发展一批优势产业。深入实施第五轮创新计划，完善科技工作推进和协调机制，稳步增加财政科技投入。加强科技创新服务体系建设，重视人才培养和引进。大力实施发明专利倍增计划，承办好第三届广西发明创造成果展览交易会。实施“质量强市”战略，积极创建全国质量强市示范城市。

十一、加强政府自身建设，提高政府的执行力和公信力

新的一年，各级政府面临着繁重而艰巨的改革发展稳定工作任务，肩负着光荣而重大的使命。我们要全面落实中央、自治区和我市关于改进工作作风、密切联系群众的各项规定，牢记“空谈误国，实干兴邦”，以实干论能力，以落实论水平，打造“清廉漓江，务实桂林”，优化发展环境，努力建设服务政府、责任政府、法治政府和廉洁政府。

坚持务实高效，提高行政效能。进一步推动政府职能向创造良好发展环境、提供优质服务、维护社会公平正义转变。坚持深入到发展最需要的地方、矛盾和问题较多的地方、群众生产生活比较困难的地方调查研究，解决实际问题。简化接待，减少繁文褥节，切实改进会风文风，开短会、开有用的会，讲短话、讲有实际内容的话，发短文、发对工作有指导推动作用的文。深化行政审批制度改革，精简审批程序，提高现场办结率和群众满意度。全面推进政府绩效管理和效能建设，大力弘扬执政为民、艰苦奋斗、不好排场、真抓实干的优良作风。

坚持科学决策，严格依法行政。自觉接受人大法律监督、政协民主监督、媒体舆论监督和社会公众监督，认真办理人大代表议案、建议和政协提案。搭建民主决策、民主管理和民主监督的有效平台，完善公众参与、专家咨询论证、社会风险评估等重大决策程序，引导社会各界人士为经济社会发展建言献策，提高政府民主决策能力。深入推进“一服务两公开”工作。

加强廉政建设，切实廉洁从政。按照党的十八大提出的“干部清正、政府清廉、政治清明”的要求，全面落实党风廉政建设责任制，切实加强审计工作，提高审计工作覆盖面、延伸度和整改率，严肃财经和资产管理纪律；切实加强行政监察，强化执行力，加大问责力度，用制度管权、按制度办事、靠制度管人。坚持厉行节约、勤俭办事，反对奢靡之风、铺张浪费，严格控制“三公”经费。深化反腐倡廉教育和廉政文化建设，严格履行“一岗双责”和廉洁自律各项规定，开展阳光纠风，加强惠农资金监督，抓好“六安工程”，严肃查处违纪违法案件，维护人民群众根本利益。

各位代表！桂林的发展已经站在了一个新的历史起点上。新的目标催人奋进，新的征程任重道远。让我们紧密团结在以习近平同志为总书记的党中央周围，深入贯彻落实党的十八大精神和自治区党委政府的决策部署，在市委的领导下，牢记全市人民的期盼和重托，牢记新的使命和责任，以更加昂扬的斗志，更加务实的作风，更加有力的举措，更加扎实的工作，锐意进取、团结拼搏，为打造国际旅游胜地、建设美丽桂林而努力奋斗！
